# Three-dimensional markerless motion capture of multiple freely behaving monkeys toward automated characterization of social behavior

**DOI:** 10.1126/sciadv.adn1355

**Published:** 2025-06-27

**Authors:** Jumpei Matsumoto, Takaaki Kaneko, Kei Kimura, Salvador Blanco Negrete, Jia Guo, Naoko Suda-Hashimoto, Akihisa Kaneko, Mayumi Morimoto, Hiroshi Nishimaru, Tsuyoshi Setogawa, Yasuhiro Go, Tomohiro Shibata, Hisao Nishijo, Masahiko Takada, Ken-ichi Inoue

**Affiliations:** ^1^ Department of System Emotional Science, Faculty of Medicine, University of Toyama, Toyama, Japan.; ^2^ Research Center for Idling Brain Science, University of Toyama, Toyama, Japan.; ^3^ Systems Neuroscience Section, Center for the Evolutionary Origins of Human Behavior, Kyoto University, Inuyama, Japan.; ^4^ Department of System Neuroscience, National Institute for Physiological Sciences, Okazaki, Japan.; ^5^ Department of Life Science and Systems Engineering, Graduate School of Life Science and Systems Engineering, Kyushu Institute of Technology, Kitakyushu, Japan.; ^6^ Center for the Evolutionary Origins of Human Behavior, Kyoto University, Inuyama, Japan.; ^7^ Cognitive Genomics Research Group, Exploratory Research Center on Life and Living Systems (ExCELLS), National Institutes of Natural Sciences, Okazaki, Japan.; ^8^ Graduate School of Information Science, Hyogo University, Kobe, Japan.; ^9^ Department of Human Intelligence Systems, Graduate School of Life Science and Systems Engineering, Kyushu Institute of Technology, Kitakyushu, Japan.; ^10^ Department of Sports and Health, Faculty of Human Sciences, University of East Asia, Shimonoseki, Japan.

## Abstract

Given their high sociality and close evolutionary distance to humans, monkeys are an essential animal model for unraveling the biological mechanisms underlying human social behavior and elucidating the pathogenesis of diseases exhibiting abnormal social behavior. However, behavioral analysis of naturally behaving monkeys requires manual counting of various behaviors, which has been a bottleneck due to problems in throughput and objectivity. Here, we developed a three-dimensional markerless motion capture system that used multi-view data for robust tracking of individual monkeys and accurate reconstruction of the three-dimensional poses of multiple monkeys living in groups. Validation analysis in two monkey groups revealed that the system enabled the characterization of individual social dispositions and relationships through automated detection of eight basic social events. Analyses of social looking demonstrated its potential for investigating adaptive behaviors in a social group. These results suggest that this motion capture system will greatly enhance our ability to analyze primate social behavior.

## Teaser

3D motion capture of multiple monkeys enables automated analysis of complex social behaviors in semi-naturalistic situations.

## INTRODUCTION

Nonhuman primates, including macaque monkeys, are social animals who use their knowledge about individual group members and their relationships to navigate a complex and dynamic social environment (*1*–*6*). Together with their evolutionary proximity to humans, this makes monkeys a vital animal model for unraveling the biological mechanisms underlying human social behavior and elucidating the pathogenesis of neuropsychiatric disorders that result in substantial difficulties in social life (*7*, *8*). However, to investigate the social functions and dysfunctions of nonverbal primates, it is necessary to assess their social interactions on the basis of body actions, which are their primary communication tools (*9*, *10*). Traditionally, behavioral actions are quantified with visual inspection by human experts. However, the substantial costs and reproducibility problems associated with manual annotation have been a major obstacle for these analyses. Recently, markerless motion capture using deep learning has been expected to overcome this issue as it improves throughput and reproducibility of quantification of various actions (*11*).

However, analysis of the social behavior of macaque monkeys in groups by using a markerless motion capture has not been achieved. Because monkeys move freely in their living environment in a three-dimensional (3D) manner, 3D tracking and pose estimation of multiple monkeys are essential for determining their social interactions. Previous reports on a primate 3D markerless motion capture system focused on the analysis of single monkeys (*12*, *13*) or only demonstrated brief analyses of interactions of a pair of monkeys as a proof of concept without describing an algorithm for multi-animal tracking (*14*). Thus, they have not been applied to the long-term behavioral analysis of groups of monkeys. Moreover, widely used deep learning frameworks for animal motion capture, such as DeepLabCut (*15*) and SLEAP (*16*), track animals in a single-view (2D), which is inappropriate for monkey groups because they move in 3D space, and frequent and severe occlusions of monkeys hinder the tracking of individuals. Recent studies in humans [e.g., (*17*)] and other animals [specifically birds, mice and dogs; (*18*–*20*)] indicate the utility of multi-view–based tracking (tracking in a 3D space) to reduce failures in individual tracking. However, these multi-view–based tracking methods have limitations regarding the separation performance of animals in contact, robustness against identity (ID) switching, or computational cost when applied to primates.

Here, we constructed a markerless motion capture pipeline using multi-camera (multi-view) data for long-term robust tracking and 3D pose reconstruction of multiple monkeys in their living environment. Applying this system to groups of Japanese macaques demonstrated that it could automatically detect eight basic social interaction events and extract their social characterization and relationships. Furthermore, quantitative motion data enabled detailed analyses of social looking, a critical component of monkey social behavior that is difficult to analyze by visual inspection (*1*, *9*, *21*).

## RESULTS

### The pipeline for the 3D motion capture of multiple monkeys

We acquired multi-view videos of monkeys in their living environment from eight synchronized cameras set in a large (4 m by 4 m) room of a semi-outdoor group cage (fig. S1). The monkeys wore a necklace-type color tag for individual identification (fig. S1B). Because the group cage had a small adjoining temperature-controlled room without cameras, the monkeys were often out of the field of view of any of the camera, thereby breaking the continuity of individual tracking (fig. S1A).

**Fig. 1.**
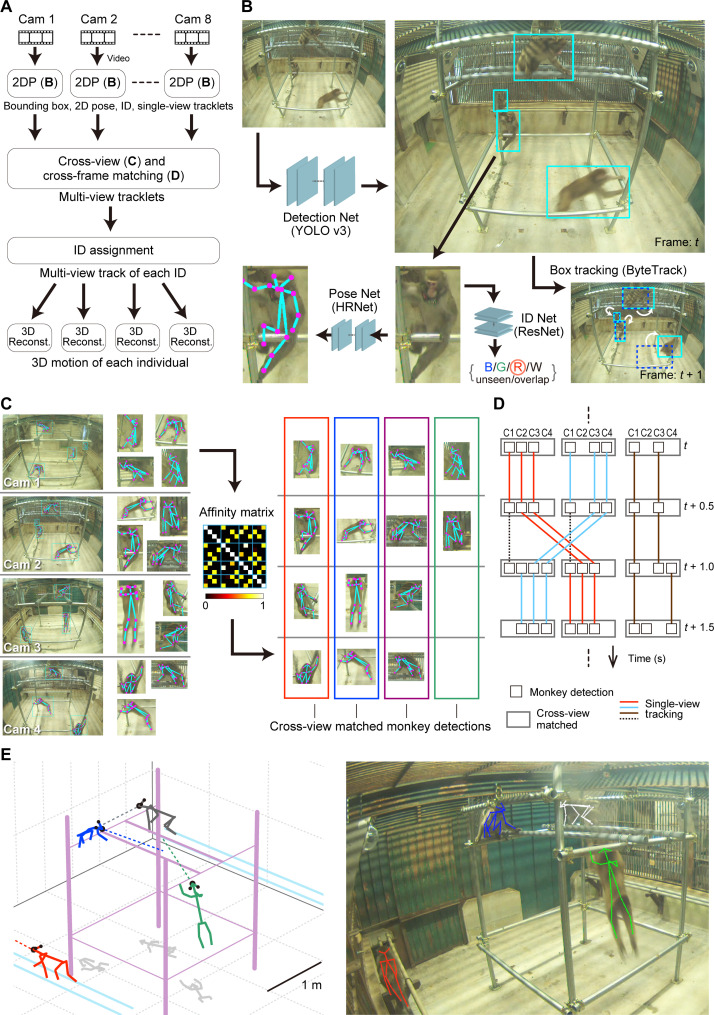
Motion capture algorithm. (**A**) Overview of the data processing pipeline. (**B**) 2D processing of a video from one camera. Three deep neural networks (Detection Net, Pose Net, and ID Net) and a box tracking algorithm were applied. The names of the algorithms are in parentheses. ID Net yielded outputs of either monkey ID [color of the tag: blue (B), green (G), red (R), and white (W)], unseen (the color tag was not detected), or overlapped (multiple monkeys overlapped in the image). (**C**) Cross-view matching. Left: Example monkey detections in each view (images from four cameras are shown for simplicity; left side, whole images; right side, zoomed images of the detections). Center: Affinity matrix representing the affinities of all pairs of detections (14 × 14 detections with the example shown in the left panel). Optimal matching was found based on the affinity matrix (*17*) (right). (**D**) Schematic of cross-frame matching. C1 to C4, cameras 1 to 4. The cross-view–matched instances (wide rectangles) were connected across frames based on single-view tracking (solid lines). The dotted lines indicate single-view tracks that were excluded because of inconsistencies. (**E**) An example of a 3D motion capture result. Left: 3D plot. Black dots indicate ears and noses. Dotted lines indicate facial direction. Line colors indicate the IDs of the monkeys. Gray lines on the floor are shadows (horizontal coordinates) of the keypoints. Right: 3D posture reprojected on the corresponding image.

The overview of the pipeline integrating multi-view information for robust multi-animal tracking is summarized in Fig. 1A. In the 2D video processing (Fig. 1B), we trained and used three deep neural networks to detect the monkeys, estimate the 2D keypoint locations (e.g., nose and knee), and identify each detected monkey in each video frame. In addition, we used a box tracking algorithm to track the monkey detections across video frames, resulting in fragments of continuous monkey tracks in the video from one camera (single-view tracklets). To integrate multi-view information, the monkey detections in different views associated with the same individuals were searched (cross-view matching) on the basis of the geometric (keypoint) and appearance affinities of pairs of detections (*17*) (Fig. 1C). Because the appearance of the monkeys used in this study was very similar, we used the ID (color tag) detections in corresponding single-view tracklets to calculate the appearance affinities between the monkey detections. To reduce computational load, we performed cross-view matching for one keyframe every 0.5 s. The connections across time between cross-view–matched monkey detection instances were estimated by maximizing the consistency of single-view tracklets (Fig. 1D). This process resulted in multi-view tracklets, i.e., sets of 2D monkey detections corresponding to the same monkey across views and video frames (colored lines in Fig. 1D). Then, the IDs were assigned to each multi-view tracklet on the basis of the results of ID detection in 2D video processing. Last, we reconstructed the 3D motion of each identified monkey from the ID-assigned multi-view tracklets using Anipose (*22*), which incorporates spatiotemporal filters into the 3D reconstruction and enables robust pose estimation compared to conventional approaches, such as combination of triangulation and temporal smoothing of 2D/3D trajectories. A representative example of 3D motion capture is shown in Fig. 1E and movie S1 (2D video processing results corresponding to movie S1 are also shown in movie S2).

### Performance validation in monkey groups

**Fig. 2.**
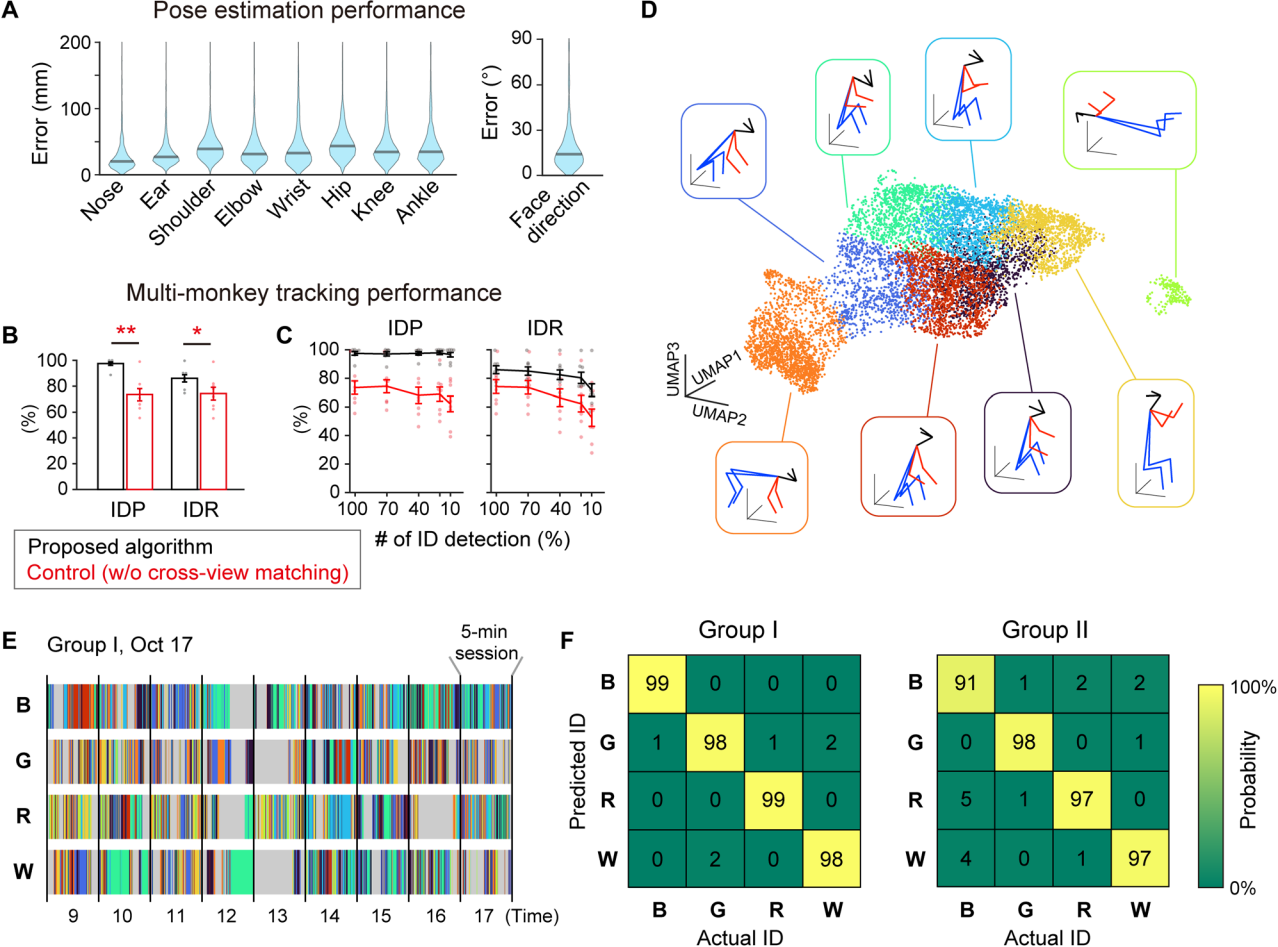
Performance validation. (**A**) Distribution of errors across 3D postures estimated by the markerless motion capture system and those by human annotation. Black line, median errors. *n* = 548 monkeys. Errors of the corresponding left and right body parts were averaged. (**B**) Mean ID tracking precision (IDP) and recall (IDR) of the proposed (black bars) and control (red bars) algorithms. Error bars indicate SEM. *n* = 8 5-min recordings. *P < 0.05; **P < 0.01, paired *t* test. (**C**) Mean IDP and IDR as functions of proportion of random reduction in the ID detection frequency. ID detection in each view was reduced by randomly masking 1-s time bins. Black and red lines indicate the performance of the proposed and control algorithms, respectively. Error bars indicate SEM. *n* = 8 5-min recordings. (**D**) Uniform Manifold Approximation and Projection (UMAP) of monkey postures. The colors of the dots indicate the result of classification using the *k*-means algorithm (*k* = 8). The inset indicates the mean posture of each cluster. (**E**) The example posture patterns of four monkeys [blue (B), green (G), red (R), and white (W)] in Group I on 1 day. A 5-min session is shown every 1 hour. Colors correspond to the classes in (D). Gray indicates that the monkey was not detected. (**F**) The confusion matrix of support vector machine (SVM) performance predicting a monkey ID based on its posture pattern.

We recorded and analyzed the social behaviors of two groups of monkeys (table S1). Each group included four monkeys wearing a blue (B), green (G), red (R), or white (W) ID tag. To evaluate the accuracy of the constructed system, we computed the errors across 3D poses estimated by the markerless motion capture and those obtained by the observation of a trained human experimenter. We assessed the errors for the keypoints and face direction, which was calculated from the locations of the nose and ears (Fig. 2A). All median errors of the keypoint locations were <50 mm, while that of face direction was 14.0°, indicating good performance in pose estimation. We also checked the system’s performance for individual tracking by comparing the estimated IDs to those detected by the human experimenter. The ID precision (IDP; correctness of estimated IDs) and recall (IDR; accuracy in recovering IDs) were 97.5 ± 1.3% and 86.0 ± 2.8% (means ± SEM), respectively, suggesting high performance. The slightly lower IDR values may be due to short tracklets without ID detection, e.g., shuttling between the recording and temperature-controlled (non-recording) rooms.

To assess the effectiveness of multi-view information in tracking, we compared its performance with a conventional approach (taken as a control algorithm), which does not use cross-view matching and does not integrate multi-view information for individual tracking (Fig. 2, B and C). The IDP and IDR of the control algorithm were lower than the values of our method on the order of 20 and 10%, respectively (Fig. 2B). Its weaker performance was partly due to erroneous integration of different monkey detections across views because of ID tracking errors (ID switch) in some views. We also tested tracking performance after randomly reducing the frequency of ID detection to check the system’s potential for applications in which ID detection will be sparser, e.g., field recordings where severe occlusions are common, or faces are used for ID instead of color tags. We found that the performance of the proposed algorithm was maintained well compared with the control algorithm [significant interaction (IDP, *P* = 0.038; IDR, *P* = 0.041) between the algorithms and the ID detection rate in two-way repeated-measures analysis of variance (ANOVA); Fig. 2C]. In addition, we found a large performance degradation when the monkey IDs were labeled only at the beginning of the videos (*19*) in both algorithms, indicating the importance of continued correction and recovery of individual tracking using ID detection and cross-view matching (fig. S2A). These results suggest our system achieved robust tracking, thanks to the utilization of multi-view information. We also found that the IDR of the proposed algorithm significantly decreased when reducing the number of views, but it maintained more than 70% in the four-camera case. On the other hand, the IDP was not affected by the number of cameras (fig. S2B). Thus, at least four cameras (preferably eight cameras) are needed for robust tracking in our setup.

To verify the practicality of this motion capture system, we attempted to identify individuals by their posture patterns. The postures were classified in an unsupervised manner using *k*-means clustering after dimension reduction (Fig. 2, D and E), and the frequency and duration of each posture were used to discriminate individuals by a support vector machine (SVM). We found that the SVM could discriminate individuals with an accuracy of >95% (98.5 ± 0.3% and 95.8 ± 1.6% in groups I and II, respectively; Fig. 2F), indicating that our system is sufficiently accurate to extract each individual’s movement characteristics.

### Automatic detection of social behavioral events and behavioral characterization

**Fig. 3.**
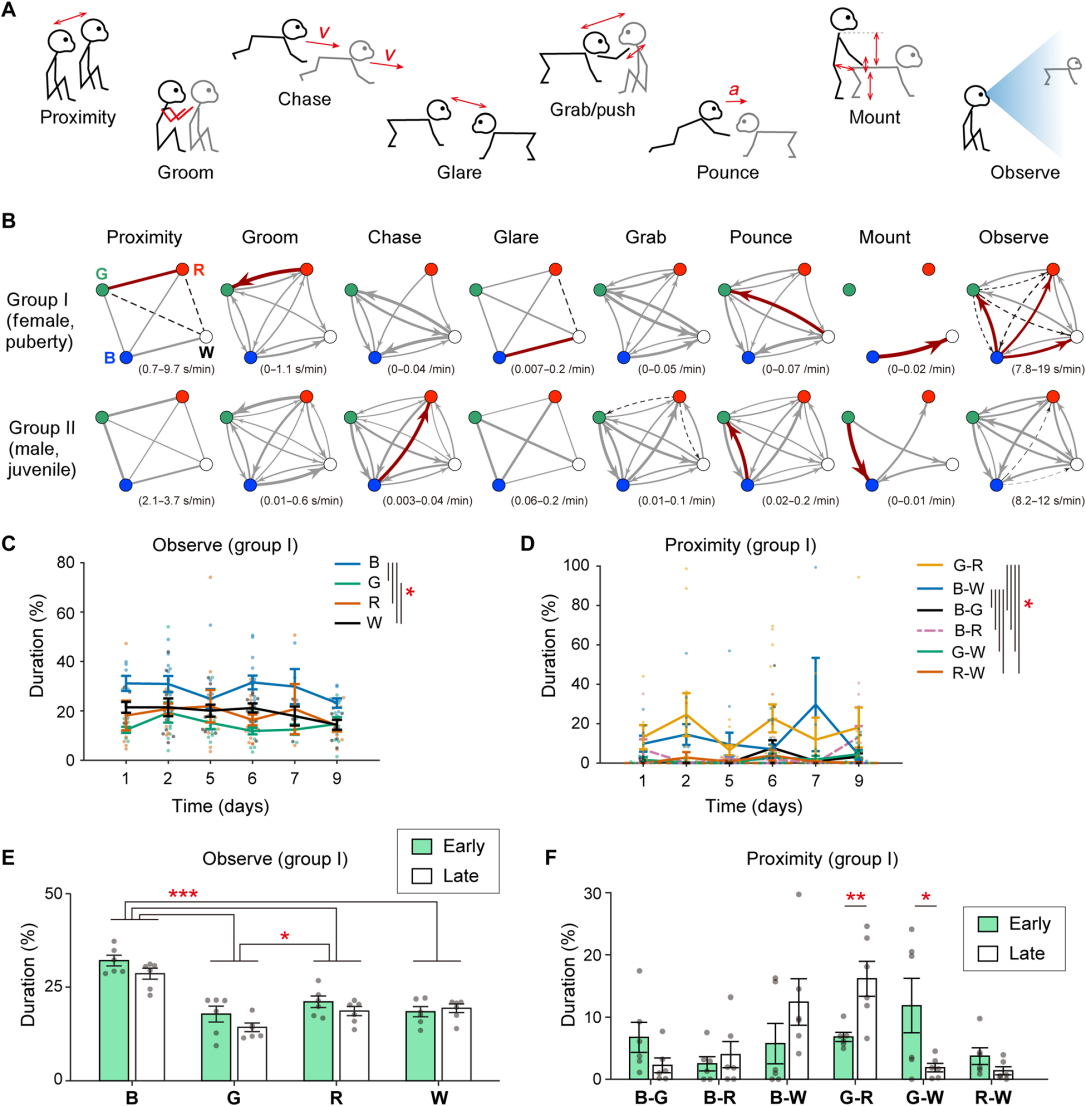
Analysis of social behavioral events. (**A**) Illustrations of definitions of social behavioral events detected automatically from the motion data. The double arrow indicates the distance between the pair. *v*, velocity; *a*, acceleration. Detailed definitions are described in Materials and Methods. (**B**) Average frequencies and durations of social behaviors between each pair of monkeys. The data are summarized as an undirected or directed graph, where each node represents an individual, and the edge thickness represents the averaged frequency or duration of the event. Red arrows and black dashed arrows indicate that the value was significantly larger and smaller than the individual shuffled data, respectively (*P* < 0.05 with Bonferroni’s correction). Parentheses indicate the ranges of values in the graphs. *n* = 58 and 111 sessions in groups I and II, respectively. (**C** and **D**) The mean duration of Observe (C) and Proximity (D) in each analysis day in group I. Days 3, 4, and 8 were excluded from the analysis since the number of available samples (recording sessions) was low (0, 1, and 2, respectively; table S2). *P < 0.05, Bonferroni’s method. *n* = 7, 11, 10, 14, 4, and 9 sessions on days 1, 2, 5, 6, 7, and 9, respectively. (**E**) Comparison of the mean duration of Observe between the early and late phases of the stay in the recording cage in group I. *P < 0.05; ***P < 0.001, Bonferroni’s method. *n* = 6 days in each phase. (**F**) A similar comparison on the mean duration of Proximity. *P < 0.05; **P < 0.01, simple main effect analysis. Error bars in (C) to (F) indicate SEM. See table S3 for the corresponding ANOVA results of (C) to (F).

Then, we analyzed the social behaviors of two groups of monkeys (groups I and II; table S1). The analyses were focused on the last eight recording days in their 3-week (group I) and 4-week (group II) stays in the group cage. Specifically, to reduce the computational load, we sampled 5-min sessions with 25-min intervals during daytime (table S2). Some sessions were excluded for technical reasons (e.g., when the experimenter was in front of the cage; see Materials and Methods for exclusion criteria). In total, 58 and 111 sessions (290 and 555 min) of groups I and II were analyzed. In this study, we defined and automatically counted affiliative (Proximity and Groom), vigorous (Chase, Glare, Grab, and Pounce), and other (Mount and Observe) social behaviors based on quantitative motion parameters, e.g., distance between two monkeys and their postures (Fig. 3A; see Materials and Methods for detailed definitions). Note that we defined the Observe as orienting the head toward a conspecific as done in previous studies (*3*, *15*, *23*) because measuring the actual gaze direction based on the eye movements of freely moving monkeys is difficult in our recording setup. Comparisons between the automatic event detections and manual event annotations by human experts are shown in Table 1 (see also movie S3 for examples of the events). These performances almost satisfied common criteria for the interobserver reliability in behavioral science (*24*) and are comparable to those of previous studies using supervised image–based behavior detection algorithms, which require “action” training data annotated manually (*25*, *26*).

**Table 1.** Social behavioral event detection performance. *n*, the total number of events detected by human experts.

	Recall	Precision	Cohen’s κ	*n*
Proximity	98%	90%	0.91	211
Groom	74%	83%	0.77	68
Chase	70%	76%	0.72	23
Glare	86%	88%	0.86	57
Grab/Push	70%	77%	0.72	33
Pounce	86%	61%	0.71	22
Mount	92%	92%	0.92	12
Observe	75%	83%	0.72	225

Automated counting of each event revealed the unique characteristics in the social disposition of individuals and groups (Fig. 3B). In the female puberty group (group I), monkey B, the smallest in the group, tended to orient her head to the other individuals more frequently. On the other hand, monkey R, which originated from a different colony than the others, tended not to participate in vigorous behavior but showed affiliative behavior to monkey G. In the juvenile male group (group II), the counts of vigorous behaviors were high, consistent with our observation that individuals in this group played frequently. Pair- or individual-specific social behaviors were fewer in group II than in group I but were still detected.

To examine the stability of the detected behavioral characteristics, we calculated the mean duration of Observe and Proximity for each analysis day in group I, in which significant individual differences of those events were found (Fig. 3, C and D). The results showed stability throughout the eight successive recording days (significant main effect of ID or pair, but no significant main effect or interaction relating to the recording date in two-way repeated-measures ANOVA; see table S3 for the ANOVA results), suggesting that the analysis could extract the social characteristics of each individual or pair. In addition, the tendency of monkey B to frequently perform Observe behavior was preserved across the initial seven recording days (early phase; 43 sessions, 215 min) and the last eight recording days (late phase) (Fig. 3E; see table S3 for the ANOVA results). Conversely, the proximity duration of the G-R and G-W pairs significantly increased (*P* = 0.0094) and decreased (*P* = 0.048), respectively, in the late phase, suggesting a proximity pair transition (Fig. 3F; see table S3 for the ANOVA results). A discriminant analysis also supported the difference in the Proximity pattern between the early and late phases (fig. S3). These results indicate that the system may be helpful in tracking long-term changes in the social relationships within monkey groups.

**Fig. 4.**
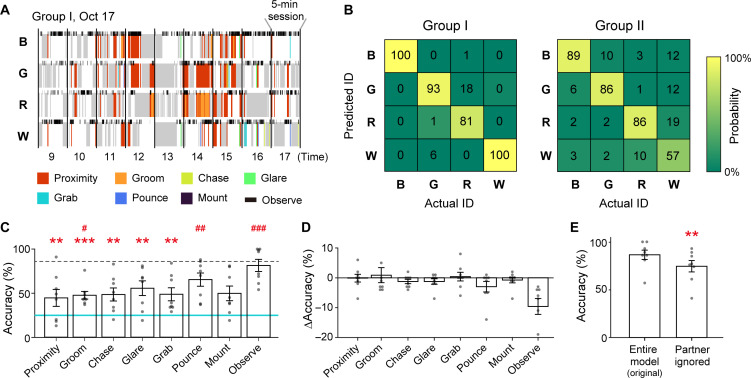
Individual discrimination based on the pattern of social behavior. (**A**) The social behavioral patterns of monkeys in a group on 1 day. The same period as Fig. 2E is shown. Colors correspond to social events. Gray indicates that the monkey was not detected. White indicates that none of the events were detected. Timings of Observe events are shown on the top of each row with narrower markers. Note that the partners of each social behavior are not presented in this graph, although partner information was used in (B) to (D). (**B**) The confusion matrix of SVM performance predicting a monkey ID based on its social behavioral pattern. (**C**) Mean prediction accuracy of SVM models using only a single type of social behavior. The dotted line indicates the mean accuracy of the entire (original) model (B). **P < 0.01; ***P < 0.001, significant difference from the entire model using a paired *t* test with Bonferroni’s correction. #*P* < 0.05; ##*P* < 0.01; ###*P* < 0.001, significant difference from the chance level (25%, solid blue line) using a one-sample *t* test with Bonferroni’s correction. *n* = 8 monkeys (all individuals in the two groups). (**D**) The mean difference in the prediction accuracy of SVM models missing a single type of social behavioral event from those of the entire model. *n* = 8 monkeys. (**E**) Comparison of the mean accuracy between the entire model and the model ignoring social behavior partners. **P < 0.01, paired *t* test. *n* = 8 monkeys. Error bars in (C) to (E) indicate SEM.

We then examined whether individuals could be discriminated from the patterns of their social behavior by SVM (Fig. 4, A and B) in the same way as with the posture patterns. The results indicated high prediction accuracy (91.8 ± 5.7% and 80.0 ± 6.4% in groups I and II, respectively), although accuracy was slightly lower than with posture patterns, especially in the male juvenile group (group II, results from a SVM trained with down-sampling of the group II dataset to that of group I are shown in fig. S4). To examine which social behavioral events were important for individual discrimination, we evaluated the performance of the SVM model when only a single type of event was used and also when a single type of event was missing. The performance of the SVM models using a single type of event (Fig. 4C) indicated that several different events could individually predict the monkey ID above chance levels, although of course performance was poorer than with the full SVM model using all events. On the other hand, leaving out the Observe event resulted in the largest drop in discrimination performance of all behavioral events (Fig. 4D). These results indicate that the social behavioral events analyzed here, especially Observe, were effective in characterizing individual monkeys. Furthermore, ignoring the ID of the social behavior partner decreased discrimination performance (Fig. 4E), indicating that social relationships were important for ID discrimination. The overall results suggest that our system has the potential to detect the social behavioral characteristics of individuals and their relationships.

### Analysis of social looking

**Fig. 5.**
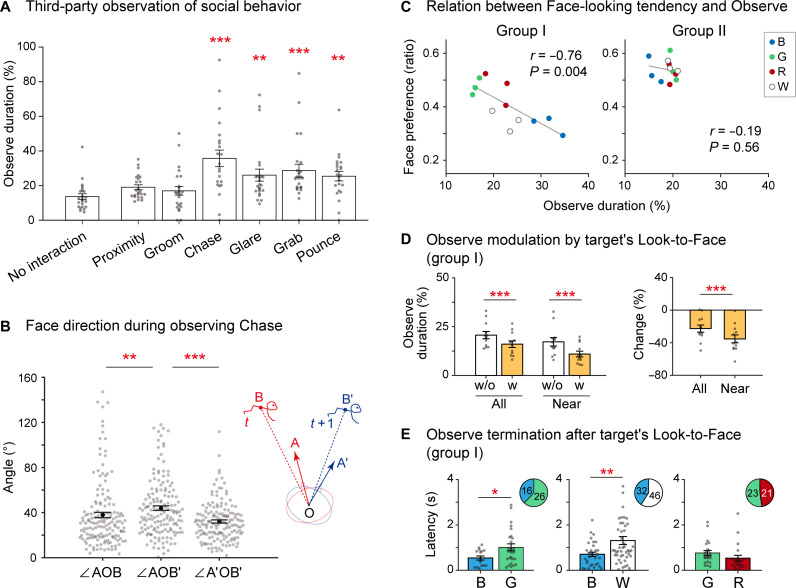
Analysis of social looking. (**A**) Mean Observe duration in 1-s time bins containing each social behavioral event by third parties. No interaction, the time bin contained no social event detection. **P < 0.01; ***P < 0.001, paired *t* test with Bonferroni’s correction comparing No interaction and the others. *n* = 24 monkey pairs from groups I and II. (**B**) Distribution of angles between third party’s face direction and target monkey’s direction during Chase. Black dots, mean angle. The inset shows an illustration of the parameters. A and A′, face directions at the beginning and end of a 1-s bin, respectively. B and B′, target monkey location relative to the third-party monkey location (O) at the beginning and end of a 1-s bin, respectively. *n* = 154 1-s bins including Chase and third party’s Observe. The data from different monkey pairs were combined. **P < 0.01; ***P < 0.001, paired *t* test with Bonferroni’s correction. (**C**) Relationship between the tendency to look around the face (Face preference index; see Materials and Methods) and Observe duration in each group. Dot colors indicate observer IDs. *n* = 12 pairs per group. *r*, correlation coefficient. (**D**) Left: Observe duration without (w/o) and with (w) the target looking to the subject’s face (Look-to-Face; see Materials and Methods) in group I. Right: Percentage changes of Observe durations by the target’s Look-to-Face. All, all conditions; Near, interindividual distance < 2 m. ***P < 0.001, paired *t* test, *n* = 12 pairs. (**E**) Latencies to terminate Observe after the target’s Look-to-Face within the three selected pairs. *P < 0.05; **P < 0.01, unpaired *t* test. Only the events in the Near condition were analyzed. The inset shows the count of Observe termination events by each monkey. There were no significant differences (binomial test, *P* > 0.05). Error bars, SEM.

Looking at a conspecific (social looking) is a critical component of monkey social behavior (*1*, *9*). We further analyzed social looking behavior. First, we compared the Observe duration calculated with and without shuffling the monkey motion data across recording sessions (fig. S5A). The actual duration of Observe behavior was significantly greater than that with the shuffled data, supporting the tendency for monkeys to observe one another. We also calculated Observe duration with temporally shifting monkey motion data (fig. S5B) and found that the peak was at zero time shift, suggesting that the observer follows the target monkey’s movement. Counting the third party’s Observe behavior toward each social behavioral event demonstrated that the monkeys tended to observe vigorous behavior by others (Fig. 5A). Furthermore, a detailed analysis of Chase behavior demonstrated that the third party’s facial direction followed the chasing movement (Fig. 5B). An unsupervised behavioral segmentation (*27*) of Observe behavior indicated that the third-parties tended to become stationary while observing vigorous behavior (fig. S6 and movie S4). Such social monitoring is essential for understanding ongoing situations and relationships between group members (*1*–*5*, *28*).

In primates, direct staring can be a threatening behavior, and gaze aversion is often associated with anxiety and submissiveness (*9*, *29*), as subordinates tend to avoid looking at the dominant’s face. In contrast, other studies reported that subordinates pay more visual attention to dominants than vice versa (*21*). Thus, it is predicted that the duration of Observe behavior may be negatively correlated with the tendency to look at the target’s face. To test this, we calculated the preference of looking near the face relative to the body (Face preference index) and examined its correlation to the Observe duration in each pair (Fig. 5C). We found a significant negative correlation in the puberty female group (group I). However, in the juvenile male group (group II), both the Face preference index and the Observe duration differed little among individuals, and the correlation was unclear. This may be related to sex/age differences in social behavior during development (see Discussion). Next, we examined whether Observe behavior is affected by the other’s gaze in group I, which showed a negative correlation in the previous analysis. We found that the duration of the Observe behavior was reduced when the target monkey was looking at the subject’s face and the two monkeys were close (Fig. 5D). A permutation test within each pair revealed that the B-G, B-W, and G-R pairs showed significant reductions of Observe behavior during the target’s gaze (fig. S7). However, in this analysis of the temporal overlap of the behavior events, which monkey in a pair caused the decrease is unclear, i.e., who is trying to avoid the mutual gaze condition. Thus, we analyzed the frequency and latency of the termination of Observe behavior after the other’s looking to the subject’s face in these pairs (Fig. 5E). We found that, in B-W and B-G pairs, monkey B, showing the most prolonged Observe duration in the group, exhibited significantly smaller latency in terminating Observe than others. These findings on the relationship between face-looking and observing behavior are consistent with the change in social looking behavior depending on the hierarchy reported in the previous studies (*9*, *21*, *29*). Together with the previous analysis in Fig. 5 (A and B), these results suggest the potential of analyzing social looking behavior based on the motion capture system for investigating monkeys’ adaptive behaviors in a social group.

## DISCUSSION

Here, we constructed a pipeline for the long-term 3D markerless motion capture of monkeys living in groups. The pipeline used multi-camera (multi-view) data for robust tracking of individual monkeys and accurate reconstruction of their 3D poses. Using this system, we obtained 3D motion data of monkeys living in groups and analyzed social behavior based on motion data. Our analysis demonstrated that this system could characterize individual motion and social traits and define their relationships. The analysis of social behavior also confirmed the findings of previous studies using human visual inspection without any post hoc manual curation on individual tracking or social behavior detection. Therefore, these results suggest that our pipeline can improve throughput and reproducibility compared to conventional social behavior analysis based on human visual inspection.

We demonstrated the system’s potential usefulness for analyzing the adaptive behaviors of monkeys in social groups through the detailed analysis of their social looking behavior, which is difficult to score manually and with high temporal resolution (*21*). We found that analysis based on the proposed system could confirm several findings from long-term observations and visual inspection by human experts in previous ecological studies (*9*, *21*, *29*). Specifically, we demonstrated that monkeys have a high incidence of observing behavior, in particular observing vigorous behavior by third parties (Fig. 5, A and B). Moreover, we found a significant negative correlation between face-looking tendency and observing behavior in the puberty female group (Fig. 5C, group I). This is consistent with the previous studies reporting a relationship between social looking and dominance hierarchy [i.e., subordinates pay more attention to dominants but avoid looking directly at the dominant’s face; (*9*, *21*, *29*)]. However, we did not find a significant negative correlation in the juvenile male group (Fig. 5C, group II). One possibility for this difference is that a clear hierarchy had not yet developed in this group. Consistent with this hypothesis, the juvenile male group showed a relatively small variance of Observe duration (SDs of group I, 6.2%; SD of group II, 1.9%; *P* = 6.0 × 10^−4^, *F* test) and Face preference index (SD of group I, 0.079; SD of group II, 0.039; *P* = 0.028, *F* test; Fig. 5C) and lower accuracy of individual prediction by SVM based on social behaviors than the puberty female group (Fig. 4B and fig. S4). Another possibility is the effect of face-to-face interaction during social play behavior. Previous studies reported that juvenile male macaques engage in social play most frequently of all sex/age combinations (*30*) and that face-to-face contact is associated with the qualities of the play (*31*). A long-term and detailed analysis of the development of social gaze in females and males will be necessary for future studies to characterize the development of sociality in monkeys.

Measuring monkeys’ 3D poses and motion in groups enabled the analysis of their various social interactions. In contrast to image classifications using supervised machine learning for detecting a specific behavior with large amounts of manually annotated training data (*25*, *26*), 3D motion data can be used flexibly to detect various behaviors, including those that are difficult to detect with image-based analysis, e.g., social looking. Previous studies also proposed 3D markerless motion capture systems for monkeys (*12*–*14*) and demonstrated detailed and automatic analysis of various behaviors of freely moving monkeys. However, these systems were mainly aimed at analyzing the behavior of single animals. Although Bala *et al*. (*14*) showed a brief proof of concept of analysis in a pair of freely interacting monkeys, in which co-occurrences of basic actions (e.g., walking, sitting, and jumping) between the pair and histogram of spatial relationships were reported, no algorithm used for multi-animal tracking was described. In addition, a single-view (2D)–based multi-animal motion capture, such as DeepLabCut (*15*) or SLEAP (*16*), can be extended to 3D by integrating estimated 2D poses of individuals in different views using a 3D reconstruction algorithm, such as Anipose (*22*). These approaches are almost identical to the control algorithm in our work, which tracks animals separately in each view (Fig. 2, B and C, red). We found that the proposed algorithm achieved more accurate and robust tracking performance than the control algorithm (Fig. 2, B and C). Thus, using the multi-view data for pose estimation and individual tracking may enable us to analyze various social interactions of monkeys in a group, which require the correct identification.

A few recent studies have also used cross-view matching to track multiple subjects in other species of nonhuman animals, such as birds and mice (*18*–*20*). However, the implementations of cross-view matching in these studies have notable differences from ours. Xiao *et al*. (*18*) generate a 3D point cloud by integrating segmentation masks (the pixels where an animal exists are white, and the others are black) of images obtained from multiple cameras. Then, each cluster of the point cloud was detected as a single animal. As the author mentioned, the method does not work well when the animals stay in contact, making multiple animals form a cluster falsely detected as a single animal. Thus, their method will be problematic when applied to a group of monkeys, as monkeys often interact more closely (e.g., grooming). Waldmann *et al*. (*19*) performed cross-view matching only once at the first frame of a video clip, and the subsequent tracking is based on the 2D tracking in each view. Thus, this method is highly vulnerable to ID switching during the 2D tracking (fig. S2A show performance of a similar algorithm with the present data). Han *et al*. (*20*) solved the cross-view matching problem by calculating the scores of all possible combinations of animal detections in different views and finding their optimal combinations. This algorithm could work for a pair of animals, but the computational cost will increase exponentially when the number of animals increases. For example, given eight cameras, the maximum number of combinations are 256, 6561, and 65,536 for two, three, and four animals, respectively. The cross-view matching algorithm implemented in our system is more efficient because the algorithm was originally designed for real-time processing (*17*). The computational cost is critical because analysis of long-term data is necessary for the characterization of social behavior. Overall, our system’s implementation of cross-view matching is optimized for long-term and robust tracking and 3D pose reconstruction of monkey groups.

Our system used an optimization algorithm for cross-view matching, but machine learning algorithms for cross-view matching have also been suggested (*32*). An important advantage of the optimization-based approach is its ease of extension. The development of 2D processing deep learning algorithms is more active than for 3D algorithms (paperswithcode.com) because of their high versatility. In addition, many existing training datasets (*14*, *33*–*36*) are available for 2D image processing. It would be relatively easy for our system to be applied to existing appropriate datasets of different species, especially for analyzing long-term social behavior in a group [e.g., (*8*, *25*)]. Moreover, updating the 2D processing algorithms used in the current pipeline to state-of-the-art algorithms would directly enhance its performance. In addition, an update of the recording hardware (e.g., higher video frame rate, higher image resolution, and more views using the latest broader bandwidth connections, such as 5-gigabit Ethernet) will enhance the utility of the pipeline for the analysis of detailed kinematics and quick movements.

Social looking is a critical component of monkeys’ social behavior and has received much attention in studies on primate social behavior. Monkeys understand the relationship between others through observations (*1*–*3*, *5*). Their visual attention toward others depends on their social relationship (*21*, *29*), and gaze and gaze aversion may be social signals by themselves (*9*, *29*). Neuroscience studies in laboratory settings have revealed the neural mechanisms involved in monkeys’ looking behavior (*6*, *7*, *37*) and their impairment in animal models of autism (*38*, *39*). Furthermore, the neural bases of sophisticated social functions in monkeys have been mainly studied in highly controlled laboratory settings while monkeys’ movement is constrained (*6*). Although these studies have provided many important insights into the neural basis of social behaviors and their dysfunctions (*6*, *7*, *37*, *40*), such approaches have problems of external validity. Examination in a more naturalistic (ethologically relevant) social environment can help to compensate for this limitation (*41*–*47*). Detailed quantitative analysis of social behavior, including social looking, of freely behaving monkeys in groups with markerless 3D motion capture will provide unique opportunities to extend findings in a specific social task in the laboratory. Because our pipeline based on 2D video processing requires only synchronized multi-camera video recording, it would be possible to construct such an analysis system with a relatively simple setup, such as attaching an imaging system to an existing group cage.

The present study has several limitations and challenges for future improvements. While this study used color tags for ID detection, our algorithm, which is robust against reductions in ID detection rate, could be adapted to use facial recognition (*46*). This would greatly enhance the utility of our system, such as in long-term monitoring of larger groups (*10*) or even in field research (*46*, *48*). However, improving robustness by larger training datasets and updating the 2D image recognition algorithms may be necessary for field research applications, as there will be increased environmental variability compared to the semi-controlled group cage environment used in the present study. Additionally, although we have successfully automated the detection of several important social behaviors (Fig. 3) and characterized some aspects of these behaviors, greater accuracy and further extensions are needed to analyze the complex and diverse social behavior in monkeys. The pipeline permitted social behavior characterization although it did not provide perfect recall and precision accuracy in behavior detection. This performance could be attributed to the fact that we are using long-term data and that the large sample provides sufficient statistical power, even if the data contain some noise due to undistinguishable/confusing behavior. However, for short-term experiments and experiments involving dynamic changes in social situations, careful confirmation of the analyzed results would be necessary because the number of samples is limited, and improved accuracy in behavior detection would be beneficial. Moreover, to cover the rich repertoire of social behaviors in monkeys (*2*, *9*, *10*), increasing types of detectable social behaviors through modalities other than body movements, such as facial expressions and vocalizations (*2*, *9*), will also be helpful. Utilization of advanced supervised (*25*, *49*–*51*) and unsupervised (*52*–*54*) machine learning for behavior segmentation using motion data and/or other types of data such as vocalizations should improve accuracy and permit more extensive detection of behavior diversity.

## MATERIALS AND METHODS

### Animals

Two groups (groups I and II) of Japanese macaques (*Macaca fuscata*) were used in this study. Each group consisted of four monkeys of the same sex and similar age (table S1). The experiments were approved by the Animal Welfare and Animal Care Committee of the Center for the Evolutionary Origins of Human Behavior of Kyoto University (2022-101) and conducted in accordance with the Guidelines for the Care and Use of Animals of the Center for the Evolutionary Origins of Human Behavior, Kyoto University.

### Video recording

A group cage consisting of one semi-outdoor large room [4 m by 4 m by 2 m (height)] and one small temperature-controlled room [2 m by 1.5 m by 2 m (height)] was used for recording (fig. S1). A small rectangular hole [0.7 m by 0.7 m (height)] with an electric door connected both rooms, making it easy for the experimenters to clean each room by keeping the monkeys in the other room. We placed eight cameras (acA2040-35gc, Basler) equipped with a wide lens (ML410 4 to 10 mm, Theia) on the wall near the ceiling inside the large room surrounding the center of the room. This camera configuration allowed most locations in the cage to be captured with ≥5 cameras (fig. S1C). Each camera was mounted in a custom stainless-steel housing with an acrylic dome window (O’Hara) securely fastened to the cage frame to prevent the monkeys from moving or touching the camera. Videos (2048 by 1536 pixels, 24 frames/sec) were captured synchronously from the eight cameras using the Motif acquisition system (Loopbio).

### Camera calibration

Intrinsic (e.g., lens distortion coefficients) and extrinsic (camera pose and location) camera parameters are required to reconstruct a 3D coordinate of a keypoint (e.g., nose, shoulder, and elbow) from 2D coordinates of the keypoint projected onto camera images from different views. To calibrate these parameters, first, we initialized the intrinsic parameters of each camera with the cv2.omnidir.calibrate() function in OpenCV (*55*) using images of checkerboards from multiple angles. Then, the extrinsic parameters of each camera were initialized with the cv2.solvePnP() function from the known 3D coordinates in the cage, e.g., corners of the room, and their 2D coordinates projected onto the camera image. Last, we waved a wand with a marker (ping-pong ball) on the tip throughout the recording volume, and the intrinsic and extrinsic parameters of all cameras were simultaneously optimized with a bundle adjustment (*22*) by minimizing the reprojection errors of the marker locations.

### Data collection

Groups I and II were moved to the recording cage and recorded for 3 weeks in October and 4 weeks across May and June, respectively. Before moving to the recording cage, the monkeys had lived with the same group members for >10 months, either in a similar-sized group cage (group I) or in a large breeding colony (group II). The monkeys wore a colored necklace for individual identification (fig. S1B). The light in the large room was turned on from 08:30 to 17:30. Food pellets were supplied once a day in the food container (fig. S1A). Supplemental fresh vegetables and fruits were given two to three times a week. Water was supplied ad libitum from water dispensers (fig. S1A). Recording was conducted daily during the daytime from 08:00 to 18:00. Sometimes, recording was paused due to technical issues. Unless noted, all data used were from each group’s last eight recording days after the monkeys were well acclimated to the recording environment.

### 2D video processing

Different deep neural network models were used for monkey detection [YOLOv3; (*56*)], pose estimation [HRNet w32; (*57*)], and monkey identification [ResNet50; (*58*)] (Fig. 1B). The monkey detection network (Detection Net) estimated bounding boxes around the monkeys in an image. The pose estimation network (Pose Net) estimated 15 keypoints (nose, left and right ears, shoulders, elbows, wrists, hips, knees, and ankles) in each cropped monkey image. The monkey identification network (ID Net) classified an ID (B, G, R, W, unknown, or monkey-overlap) of each cropped monkey image. We used B, G, R, and W classifications with confidence values of >0.9 in the following processes. To train and test the Detection Net and Pose Net, 2438 (training, 2238; test, 200) images captured in the experiments, including 6435 monkeys (training, 5927; test, 508), were manually annotated by Cocosnet Ltd. We developed a custom annotation software capable of multi-view augmentation (*14*) and reprojected 3D keypoint positions to images of all eight camera views (movie S5). Note that we pretrained Pose Net with the MacaquePose dataset (*26*), containing 13,083 images (16,393 monkeys) captured in various naturalistic contexts. For training and test of ID Net, 16,510 (training, 16,270; test, 240) and 2023 (training, 1904; test, 119) cropped monkey images for groups I and II, respectively, were manually labeled by a trained experimenter. The average precision of the trained neural networks was 0.744 for Detection Net, 0.735 for Pose Net, 0.853 for ID Net for group I, and 0.810 for ID Net for group II. In addition, bounding boxes were tracked in a single-camera video using the ByteTrack algorithm (*59*), resulting in tracklets, i.e., fragments of monkey tracks. Here, we call this type of tracklets generated with each camera view as single-view tracklets, while we call tracklets generated by integrating information from all views in the way described later as multi-view tracklets. We built this 2D video processing pipeline using popular computer vision libraries provided by OpenMMLab (openmmlab.com).

### Multi-view tracklet generation

We generated multi-view tracklets, i.e., a set of 2D monkey detections (estimated in the process described above) corresponding to each individual across views and video frames by the following processing. For cross-view matching of 2D monkey detections, we customized and used the MVPose algorithm suggested for human pose estimation (Fig. 1C) (*17*). To estimate optimal cross-view matches, we calculated the affinity matrix (**A**), which represents the affinity of all pairs of 2D detections in all views in a time point. We defined the affinity matrix as a weighted sum of geometric affinity (**A**^g^) and appearance affinity (**A**^a^), as follows

$$A_{\mathrm{ij}}=\alpha A_{\mathrm{ij}}^{g}+\left(1-\alpha \right)A_{\mathrm{ij}}^{a}$$ (1)

where *i* and *j* are an index of a pair of 2D monkey detections (1 ≤ *i*, *j* ≤ *m*; *m*, the total number of 2D monkey detections), and α is constant [0.8 in this study; in the original MVPose for humans (*17*), α was 0.2. In this study, we tended to use geometric affinity because the appearance affinity of monkeys is less reliable than that of humans wearing different clothes]. To derive geometric affinity (**A**^g^), we calculated the geometric distance (**D**) of a pair of 2D monkey detections as follows

$$D_{\mathrm{ij}}=\frac{1}{N}\sum_{k=1}^{N}d_{\mathrm{ijk}}$$ (2)

where *N* is the number of keypoints and *d_ijk_* is the distance between camera rays to the *k*th keypoint of the *i*th and *j*th 2D monkey detections. Only the keypoints commonly detected (prediction confidence of >0.1) in the *i*th and *j*th 2D monkeys were included. Then, we defined geometric proximity (**C**) as follows

$$\begin{array}{c} C_{\mathrm{ij}}=\beta -D_{\mathrm{ij}},\text{if}\;D_{\text{ij}}<\beta \;\\ C_{\mathrm{ij}}=0,\;\text{otherwise}\; \end{array}$$ (3)

where β is constant (1500 mm in this study). The geometric affinity (**A**^g^) was obtained by mapping the proximity **C** to values in (0, 1) with a sigmoid function. The original algorithm calculated the appearance affinity (**A**^a^) using image feature values obtained with a person re-ID network (*60*), which extracts view-invariant discriminative appearance features such as clothing and hairstyle. However, extracting such view-invariant discriminative appearance from similar-looking monkeys is difficult, so we estimated a putative ID of each single-view tracklet on the basis of the outputs of the monkey identification network with a similar ID assignment algorithm used for the multi-view tracklets (see the next section) applied separately to each view. Then, we calculated appearance affinity (**A**^a^) as follows

$$\begin{array}{c} A_{\mathrm{ij}}^{a}=1,\text{if}\;{\text{pID}}_{i}={\text{pID}}_{j}\\ A_{\mathrm{ij}}^{a}=0,\text{otherwise}\; \end{array}$$ (4)

where pID*_i_* represents the putative ID of the single-view tracklet containing the *i*th detection at the time point.

Last, the permutation matrix (**P**), which represents the matching of all detection pairs, was estimated. The optimal permutation matrix should maximize the affinity and be cycle consistent, i.e., in a matched group, there is no more than a single detection per view. According to Dong *et al*. (*17*), such a permutation matrix was estimated by solving the following optimization problem with the alternating direction method of multipliers

$$\begin{array}{c} \underset{P}{\text{min}}-\langle A,P\rangle +\lambda {\left\|P\right\|}_{*}\\ s.t.\;P\in C \end{array}$$ (5)

where <**A**, **P**> denotes the inner product of the matrices, λ represents a constant (50 in this study), and ${\left\|P\right\|}_{*}$ represents the nuclear norm of **P**. *C* represents a set of matrices satisfying the following constraints

$$\begin{array}{c} 0\leq P\leq 1,\\ P=\left(\begin{array}{ccc} P_{11} & \cdots & P_{1n}\\ \vdots & P_{\mathrm{vw}} & \vdots \\ P_{n1} & \cdots & P_{\mathrm{nn}} \end{array}\right) \end{array}$$ (6)

where *n* is the number of views and **P***_vw_* is a permutation matrix that represents the matching of detection pairs of view *v* and *w*. **P***_vw_* satisfies the following constraints

$$\begin{array}{c} 0\leq P_{\mathrm{vw}}1\leq 1,\;0\leq P_{\mathrm{vw}}^{T}1\leq 1\\ P_{\mathrm{vw}}=P_{\mathrm{vw}}^{T},1\leq v,w\leq n,v\neq w\\ P_{\mathrm{vv}}=I,\;1\leq v\leq n \end{array}$$ (7)

This cross-view matching was performed for one keyframe every 0.5 s to reduce computational load. Between the keyframes, cross-view and cross-frame matching was estimated using single-view tracklets and sets of the cross-view–matched 2D monkey detections at keyframes. Specifically, we defined the tracking consistency of a pair of sets of the matched 2D monkey detections in neighboring keyframes as the number of single-view tracklets shared by both sets (Fig. 1D). Then, matching of the pairs of the sets that maximized the total tracking consistency was calculated using the Hungarian method, resulting in multi-view tracklets, i.e., a set of 2D monkey detections corresponding to an individual monkey across views and video frames.

### ID assignment to multi-view tracklets

Then, the ID of each multi-view tracklet was estimated. To compensate for occasional errors of the monkey identification network, first, we defined a detected ID of a multi-view tracklet to be valid if it passed the following criteria in a time window

$$N_{l_{\text{max}}}\geq 12,\;N_{l_{\text{max}}}/\;\sum_{l}N_{l}>0.8$$ (8)

where *l* represents an index of ID (B, G, R, or W), *l*_max_ represents an index of the most detected ID, and *N_l_* is the detection count of ID *l*. The valid ID of each time point was checked using a 5-s sliding window. If only a single valid ID was found in a multi-view tracklet, the ID was assigned to the tracklet. If multiple valid IDs were found, then the tracklet was separated at the midpoint when different valid IDs were found. If no valid ID was found, then Eq. 8 was reexamined with a time window covering the entire time range of the tracklet, and, if it passed, then the ID was assigned to the tracklet.

Because the linking of multi-view tracklets was interrupted when no correspondence was found between the neighboring keyframes, e.g., in the case of severe occlusion, second, we stitched the tracklets together on the basis of their continuity using network flow optimization (*15*, *61*). Specifically, a directed graph consisting of nodes representing tracklets and edges representing possible connections between tracklets was constructed. A pair of multi-view tracklets (nodes) separated by no more than 5 s and having at least one common single-view tracklet were connected with an edge. Each edge had a cost value equal to the distance between the monkey’s neck location at the end of one tracklet and the beginning of the other tracklet. If the assigned IDs of a tracklet pair were different, then the corresponding edge was removed. If the IDs were the same, the cost value of the corresponding edge was divided by 100. In addition, sink and source nodes were assumed and connected to all tracklet nodes by the edges of a fixed amount of cost (1000 mm in this study). Then, optimal associations between tracklets were calculated by a minimum-cost flow algorithm using the capacity_scaling() function in the NetworkX library (networkx.org), and the tracklets were stitched accordingly.

Third, the IDs were reassigned to the stitched tracklets using the same criteria (Eq. 8). Sometimes, the ID assignment resulted in multiple tracklets having the same ID at the same time. We resolved such ID overlaps as follows: (i) if an overlapping tracklet was previously stitched and the original (before stitching) tracklet corresponding to the overlapping part had no valid ID, then the tracklet was unstitched; (ii) overlapping tracklets without a nonoverlapping period (fig. S8A) was excluded; and (iii) overlapping tracklets were trimmed on the basis of the timings when the valid IDs were detected (fig. S8B). Last, the tracklets that had no ID yet and that there were ≥3 frames in which tracklets with the other three IDs were present during the period of the tracklet were assigned to the fourth (final) ID.

### Pose filtering

We reconstructed the 3D motion of each monkey using the multi-view tracklets with the corresponding ID. For robust 3D motion estimation, we used the Anipose algorithm (*22*). Briefly, 2D keypoint trajectories were Viterbi filtered in each view, and, then, the reasonable 3D motion of an animal was estimated by minimizing the total of the following three losses: the error of body part length, change in keypoint acceleration (third derivative of the trajectories), and reprojection error. The total loss was calculated by summing each loss after scaling (scaling factors were 10, 10, and 1 for the body part length error, the third derivative of the trajectories, and reprojection error, respectively). For the 2D Viterbi filtering, we used the default parameters of Anipose (version 1.0.1). For the 3D motion estimation with Anipose, we changed the default setting that optimizes the smoothness of the velocity to the setting that optimizes the smoothness of the acceleration to preserve detailed movements.

### Detection of social behavioral events

We calculated basic behavioral parameters using the estimated 3D motions of monkeys to detect behavioral events. Specifically, we determined the position (neck; midpoint between left and right shoulder), speed, and face direction (vector from the midpoint of the left and right ears to the nose, rotated upward by 35°) of each monkey. We also calculated the distance and approaching/leaving speed (speed along the axis connecting the pair) for each pair of monkeys. We low-pass–filtered these parameters with a cutoff frequency of 0.5 Hz. In addition, we classified body-centered postures in a feature space obtained with principal component analysis to detect sitting, lying (fig. S9A), and typical forelimb posture associated with grooming (fig. S9B). The social behavioral events were detected (Fig. 3A) with these parameters according to the following definitions. In the following definitions, for directional behavioral events, we call the subject of the behavior as monkey X and the object as monkey Y.

#### Observe

The angle between the face direction of monkey X and a vector from the nose of monkey X to the body center (mean coordinate of left and right shoulders and hips) of monkey Y is <40°. The monkey X (observer) does not move much (speed of <500 mm/s).

#### Proximity

The monkey pair shows a sitting or lying posture (fig. S4A) and do not move fast (speed of <1500 mm/s). The distance between the monkeys is <600 mm.

#### Grooming

Monkey Y is either sitting or lying. Monkey X is in one of the grooming-associated postures (fig. S4B) corresponding to the posture of monkey Y. The distance between the monkeys is <500 mm, and neither monkey moves fast (speed of <1500 mm/s). The angle between the face direction of monkey X and a vector from the nose of monkey X to the neck of monkey Y is <70°, i.e., monkey Y is in front of monkey X.

#### Mount

The distance between the hips of the monkeys is <300 mm. The relative neck height of monkey X from that of monkey Y is >200 mm. The relative wrist height of monkey X from the neck of monkey Y is <200 mm. The mean distance between the wrists of monkey X and the neck of monkey Y is <400 mm. The relative ankle height of monkey Y from the neck of monkey Y is <−150 mm. Neither monkey is in a sitting or lying posture.

#### Chase

Monkey X approaches monkey Y (approaching speed of >1000 mm/s), while monkey Y leaves from monkey X (leaving speed >1000 mm/s). Both monkeys move fast (speed of >1500 mm/s). The distance between the monkeys is <2500 mm.

#### Glare

The monkeys are looking at each other (the face direction of monkey X and a vector from the nose of monkey X to the neck of monkey Y is <40° and vice versa). The distance between the monkeys is <1500 mm. Neither monkey moves fast (speed of <1500 mm/s). At least one of the monkeys is not sitting or lying.

#### Grab/Push

The distance from either of monkey X’s wrists to the neck of monkey Y is <200 mm. The distance between the monkeys is >350 mm. The posture of monkey X is not sitting or lying. Monkey X is not mounting monkey Y.

#### Pounce

The distance between the monkeys is <600 mm. Neither monkey moves fast (speed of <1500 mm/s). At least one of the monkeys is not sitting or lying. The approach speed from monkey X to monkey Y is >0 and <1500 mm/s. The acceleration of the approach speed is >2500 mm/s.

For the event detections, first, the frames that satisfy the above criteria were marked. Then, the event markers were filtered according to the following rules: If the same type of event occurred within an interval of less than *I*_max_, then the event was considered to have continued during the interval; and if the duration of an event was less than *D*_min_, then the event was ignored. The filter parameters for each event are shown in table S4.

To examine whether monkey X prefers looking to the face or the body of monkey Y, we defined the Face preference index as *T*_Face_/(*T*_Face_ + *T*_Body_), where *T*_Face_ represents the total time that monkey X looks closer to the face than to the body, and *T*_Body_ represents the total time that monkey X looks closer to the body than to the face. To ensure the attention to either part of the body, we counted *T*_Face_ and *T*_Body_ only when (i) monkey X does not move much (speed of <500 mm/s; as in Observe definition); (ii) the face of monkey Y can be seen from monkey X (the angle between the face direction of monkey Y and a vector from the nose of monkey Y to the nose of monkey X is <60°); (iii) the angle between the face direction of monkey X and a vector from the nose of monkey X to either the body center or the nose of monkey Y is <30°; and (iv) the distance between monkey X and monkey Y is <2 m. We also defined the Look-to-Face behavior event using similar criteria to the Face preference index, as follows: (i) The angle between the face direction of monkey X and a vector from the nose of monkey X to the nose of Monkey Y is <30°; (ii) monkey X does not move much (speed of <500 mm/s). The Observe termination after the target’s Look-to-Face (Fig. 5E) was counted when the subject began Observe before the target’s Look-to-Face and ended Observe during the target’s Look-to-Face. Because the monkeys were smaller in group II than in group I, the distance, speed, and acceleration thresholds above were normalized by multiplying the values by 0.82 for group II.

### Performance validation

To examine the performance of 3D pose estimation, we annotated the 3D poses of monkeys with one frame every 12 s from eight 5-min video clips that did not overlap with the training dataset. The error of each keypoint between a manually annotated and automatically estimated pose was calculated when the corresponding instance was found (root mean square error of all keypoints of <500 mm). To assess the performance of 3D tracking, we annotated the 3D neck positions of monkeys with IDs in 2.5 frames/s from the same video clips. Manually annotated instances and automatically estimated instances in each frame were matched by the Hungarian method to minimize the total distance between the necks of the matched pairs. If the distance of a matched pair was <400 mm and the IDs of the pair were coincident, then it was counted as successful [true positive (TP)]. The number of false positives (FPs) and false negatives (FNs) was calculated by subtracting TP from the total number of automatically estimated or manually annotated instances, respectively. To assess the effectiveness of multi-view information in tracking, we compared the proposed algorithm with a similar one in which the “multi-view tracklet generation” part was removed (the control algorithm). In the control algorithm, the ID assignment to single-view tracklets was performed in each view similarly as described in the “ID assignment to multi-view tracklets” section. For the tracklet stitching, the cost values of the edges were calculated as the distance in the image (in pixels) instead of the absolute distance (in millimeters). The costs of the edges from the tracklet nodes to the sink and from the source to the tracklet nodes were set to 500 pixels.

We tested the performance of social behavioral event detection using another dataset. Specifically, two human experts annotated the occurrence and direction (from which monkey to which monkey) of all defined social behavioral events (Fig. 3A) except for Observe between a given pair in 10-s video clips, and annotations common to both experts were used as the ground truth. TP, FP, FN, and true-negative (TN) cases were counted by comparing the annotated results with the automatic estimation. Eight hundred and seven video clips were selected on the basis of successful tracking (both monkeys were tracked for >50% of the time of the clip) and potential of interaction (the distance between the monkeys was <1 m at least once in the clip) and were presented to human experts for annotation. For short events, i.e., Grab/Push and Pounce, video clips were excluded from counting if there was any brief interruption in monkey detection during the event. Because Mount was a rare event, we used 3001 video clips selected in the same way. To prepare the ground-truth data for Observe, as it is very difficult for humans to determine a monkey’s face direction from 2D videos accurately, we combined the monkeys’ face directions and locations computed from the keypoints manually annotated for the 3D pose estimation validation described above with additional manual annotations of whether or not the monkeys were moving at the corresponding video frames.

We defined and calculated precision and recall values as TP/(TP + FP) and TP/(TP + FN), respectively, to evaluate the performance of ID tracking and behavioral event detection. Cohen’s κ values (*24*) were also calculated for the performance of behavioral event detection, as follows

$$\kappa =\frac{p_{o}-p_{e}}{1-p_{e}}$$ (9)

where *p*_o_ and *p*_e_ are an observed agreement ratio and an agreement ratio expected by chance, respectively. Specifically, *p*_o_ and *p*_e_ were calculated as follows

$$\begin{array}{c} p_{o}=\left(\text{TP}+\text{TN}\right)/N_{c}\\ p_{e}=\text{[TP}\times \left(\text{TP}+\text{FN}\right)/N_{c}+\text{TN}\times \left(\text{TN}+\text{FP}\right)/N_{c}]/N_{c} \end{array}$$ (10)

where *N*_c_ is the total number of examined video clips.

### Behavioral data analysis

The behavioral data analyses were mainly focused on 08:30 to 17:30 on each group’s last eight recording days. We sampled 5-min sessions with 25 min intervals (08:30 to 08:35, 09:00 to 09:05,…, 17:00 to 17:05) for the analysis to reduce computational load. We excluded a session from the analysis if an experimenter was inside or in front of the cage. Only sessions with >30-s detection of all four monkeys were used to analyze their interactions. See table S2 for the list of sessions that passed the criteria.

We used an SVM to predict monkey IDs from behavioral patterns to assess individual differences in the behavioral patterns. Twenty sessions (100 min) were combined and used as input to the SVM, and leave-one-out cross-validation was used to evaluate the performance of the SVM. Specifically, we used a set of 20 randomly sampled sessions as test data. From the rest of the data, we randomly sampled 100 combinations of 20 sessions and used them for training the SVM. Then, we compared the trained SVM’s ID predictions on the test data with the correct IDs. This test was repeated 100 times with differently sampled test and training data, and the results of all the repetitions were summed up to create the confusion matrix (Figs. 2F and 4B and fig. S4A) of the ID prediction by the SVM.

### Statistical analysis

Basic statistical methods such as one sample *t* tests, binomial tests, paired *t* tests, and repeated-measures ANOVA were used for statistical comparisons. Bonferroni’s correction was used for multiple comparisons. The statistical tests were performed using SPSS and MATLAB. The significance threshold was set to 0.05. The values for *N* and *P* and the specific statistical test performed for each experiment are included in the corresponding figure legend, Results, or Discussion.
