# Supplementary material for: Three-dimensional markerless motion capture of multiple freely behaving monkeys toward automated characterization of social behavior
Source: Sci Adv. 2025 Jun 27;11(26):eadn1355. doi: 10.1126/sciadv.adn1355 (PMC12204171; doi:10.1126/sciadv.adn1355)
Supplement: Supplementary file 1 — Figs. S1 to S9 Tables S1 to S4 Legends for movies S1 to S5 [file sciadv.adn1355_sm.pdf]

Science Advances
AAAS

Supplementary Materials for

Three-dimensional markerless motion capture of multiple freely behaving monkeys toward automated characterization of social behavior

Jumpei Matsumoto *et al.*

Corresponding author: Jumpei Matsumoto, jm@med.u-toyama.ac.jp; Takaaki Kaneko, kanekot@nips.ac.jp; Ken-ichi Inoue, inoue.kenichi.6z@kyoto-u.ac.jp

Sci. Adv. **11**, eadn1355 (2025)
DOI: 10.1126/sciadv.adn1355

The PDF file includes:

Figs. S1 to S9
Tables S1 to S4
Legends for movies S1 to S5

Other Supplementary Material for this manuscript includes the following:

Movies S1 to S5

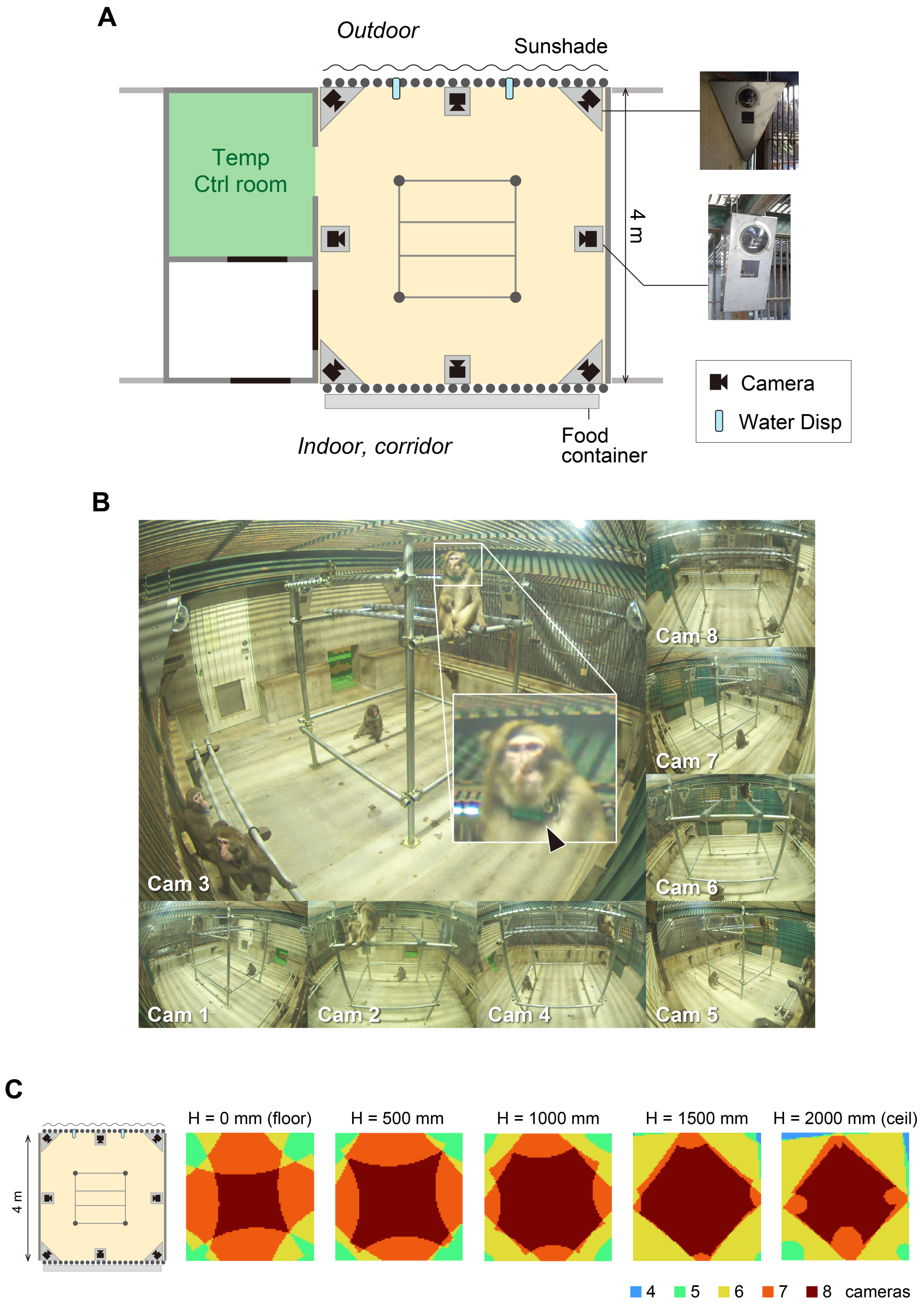

Fig. S1. Recording setup

(**A**) Schema of the recording setup. The square in the center of the cage represents a jungle gym. Temp Ctrl room, temperature-controlled room. Water Disp, water dispenser. Inset pictures show the camera housings. (**B**) An example set of images captured simultaneously from the eight cameras. The inset shows the color tag (arrowhead) for monkey identification. (**C**) Overlap of the camera views at each location within the cage volume. The color indicates the number of cameras capturing the location. H, height. Note that the occlusions by the objects in the cage and monkeys are not considered in this figure.

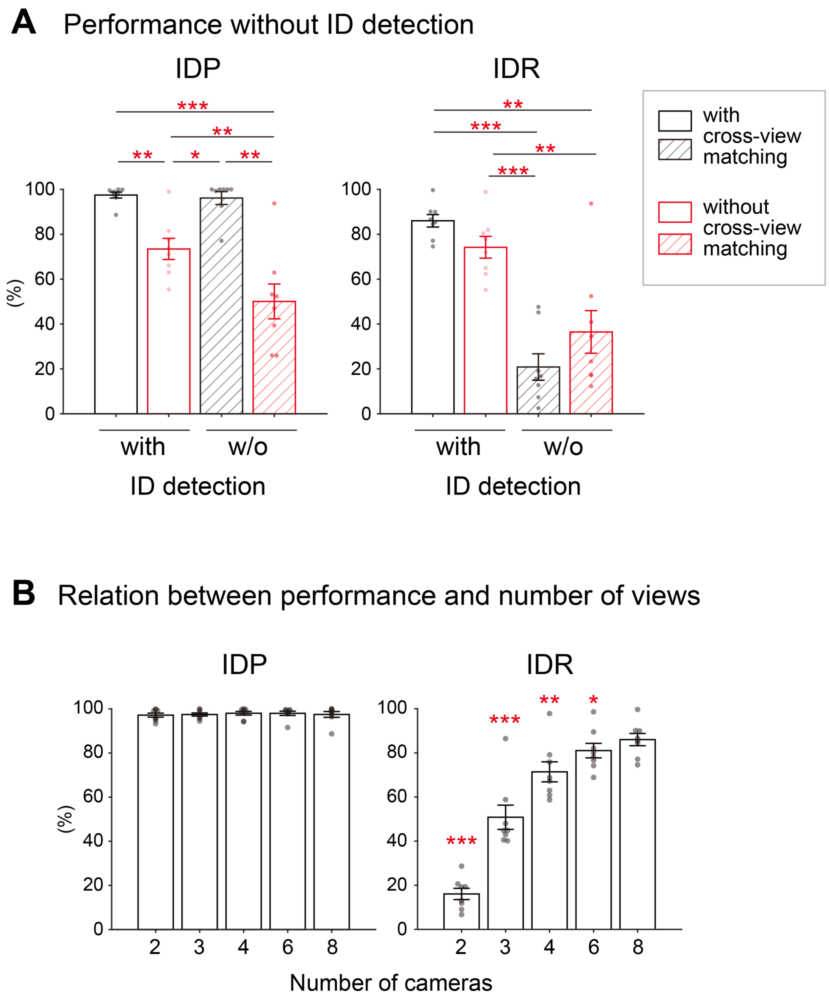

Fig. S2. The additional performance validation with different conditions.

(**A**) The tracking performance of the algorithms without ID detection. The open and striped bars represent the algorithms with and without ID detection, and the black and red bars represent the algorithms with and without cross-view matching, respectively. For the algorithms without ID detection, we manually labeled the ID of each monkey's cross-view matched detections only at the beginning of each 5-min recording session. The black and red open bars are the same as the proposed and the control algorithms in Figure 2B, respectively. Note that the algorithm without ID detection and cross-view matching (the red striped bar) is similar to the algorithm proposed by Waldmann et al. (19). For the algorithm without ID detection + cross-view matching only at beginning (purple, the algorithm similar to Waldmann et al. (19)), the cross-view matching was performed only once at the beginning of the video clip, and the ID of each monkey was manually assigned to a group of matched 2D monkey detections. The subsequent tracking is based on the 2D tracking in each view. ***, $p < 0.001$, **, $p < 0.01$, *, $p < 0.05$, paired t-test with Bonferroni's correction. (**B**) Relationship between the tracking performance and the number of views (cameras). Performance was measured with different numbers of cameras by excluding some of the views from the analysis. *$p < 0.05$, **$p < 0.01$, ***$p < 0.001$, paired t-test compared with the value of the eight-camera condition with Bonferroni's correction. Other descriptions are the same as in Fig. 2B.

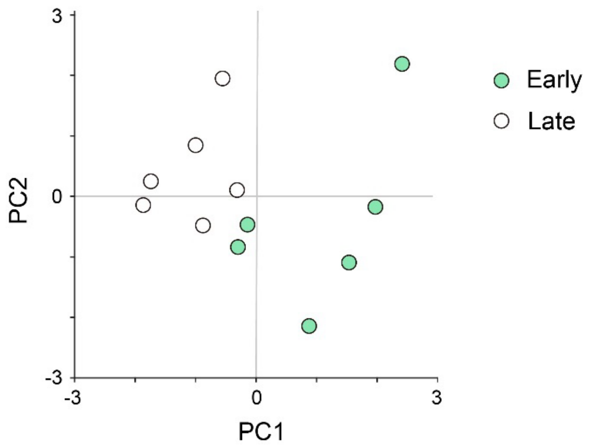

Fig. S3. Comparison of proximity patterns in the early and late phases of Group I.

Each point represents the pattern of Proximity one recording day. The Proximity pattern was calculated as a vector with six elements, where the elements represent the mean Proximity duration for the six pairings of the four monkeys. The dimensions of the proximity pattern were reduced to two dimensions (PC1 and PC2) by principal component analysis. Discriminant analysis indicated significant separation between the points of the early and late phases (Wilks' lambda = 0.248, p = 0.002, n = 6 days for each phase).

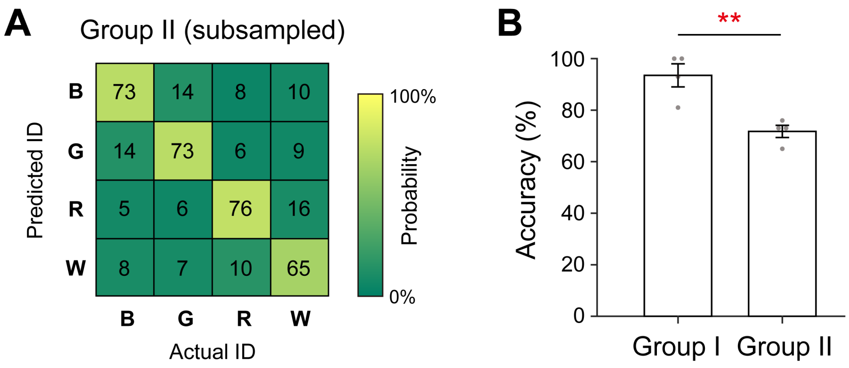

Fig. S4. Accuracies of the predicted IDs by SVMs in Fig. 4 after subsampling.

(**A**) For an equitable comparison, the number of sessions used for training the SVM of Group II was down-sampled (from 111 sessions) to that of Group I, by randomly selecting 58 recording sessions (= number of sessions of Group I) at the beginning of each iteration of the cross-validation. The resultant confusion matrix of the SVM with the subsampling is shown. (**B**) Comparison of the ID prediction accuracy of the SVMs of Group I and Group II with subsampling. $^{**}p < 0.01$, unpaired t-test. $n = 4$ monkeys for each group.

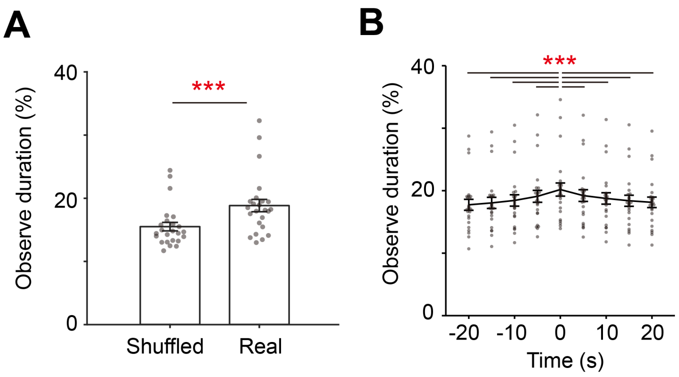

Fig. S5. The pattern of the Observe behavior is not random.

(**A**) Comparison of the mean Observe duration with (Shuffled) and without (Real) shuffling of the subjects' motion data across recording sessions. ***$p < 0.001$, paired *t*-test. $n = 24$ pairs of monkeys from Groups I and II. (**B**) Mean Observe duration with temporally shifting subjects' motion data. Horizontal axis, the amount of time shift. ***$p < 0.001$, paired *t*-test compared with the value at time = 0 with Bonferroni's correction. $n = 24$ pairs of monkeys from Groups I and II.

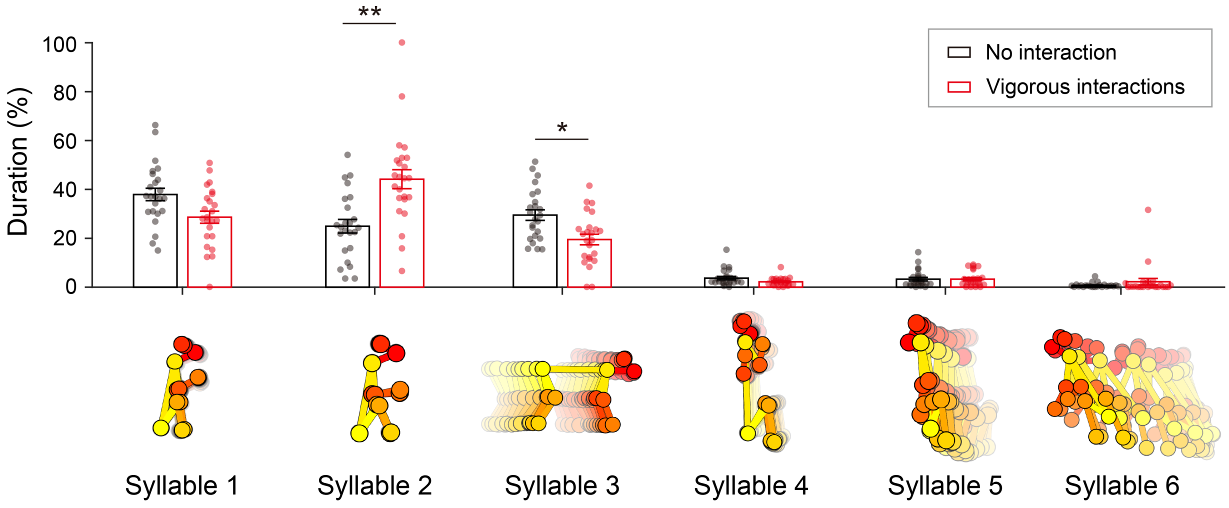

Fig. S6. Analysis of action motif of third-party observers with unsupervised behavioral segmentation.

Using Keypoint-MoSeq (27), the motion data during Observe were classified into six action syllables: sitting with a little movement (Syllable 1), sitting with almost no movement (Syllable 2), slow locomotion (Syllable 3), more dynamic movement around sitting (Syllable 4), and turning (Syllable 5 and 6). This figure shows the mean duration of each syllable by third-party observers while they observed a monkey that was having no detected social interactions with another monkey (black) and a monkey engaged in vigorous interactions (Chase, Glare, Grab, and Pounce) with another monkey (red). The significant increase of Syllable 2 and decrease of Syllable 3 suggests that the monkeys were more stationally while observing the vigorous interaction. *, **, $p < 0.05$, 0.01, paired t-test with Bonferroni's correction. $n = 24$ pairs of monkeys from Groups I and II. In the classification using Keypoint-MoSeq, the default parameters were used except for the stickiness hyperparameter $\kappa = 10^{10}$ during the full model fitting. See Movie S4 for examples of actions classified as Syllable 1 and 2.

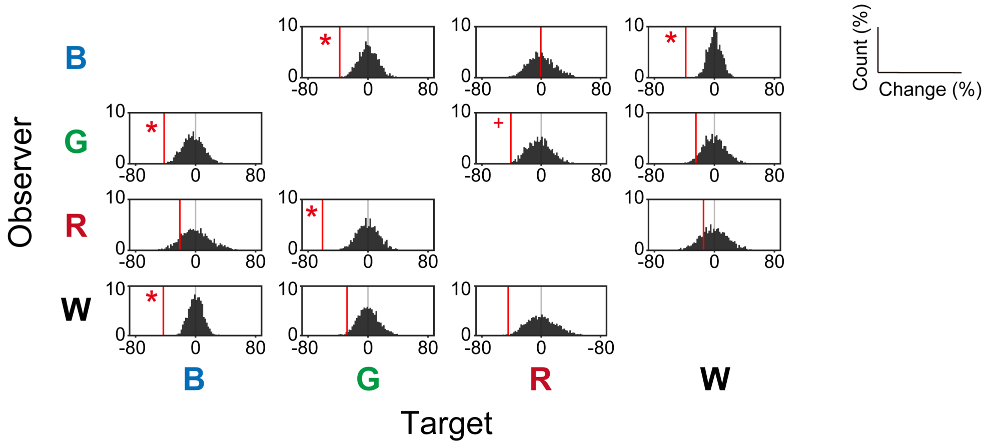

Fig. S7. Change of Observe duration associated with the target's looking at the subject's face in each pair in Group I.

The actual value (red line) and chance distribution (gray histogram) of the percentage change (Fig. 5D, right) were calculated for each pair. The chance distribution was obtained by shuffling Observe event timings (n = 2,000 repetitions). $^{+}p < 0.1$, $^{*}p < 0.05$, permutation tests with Bonferroni's correction.

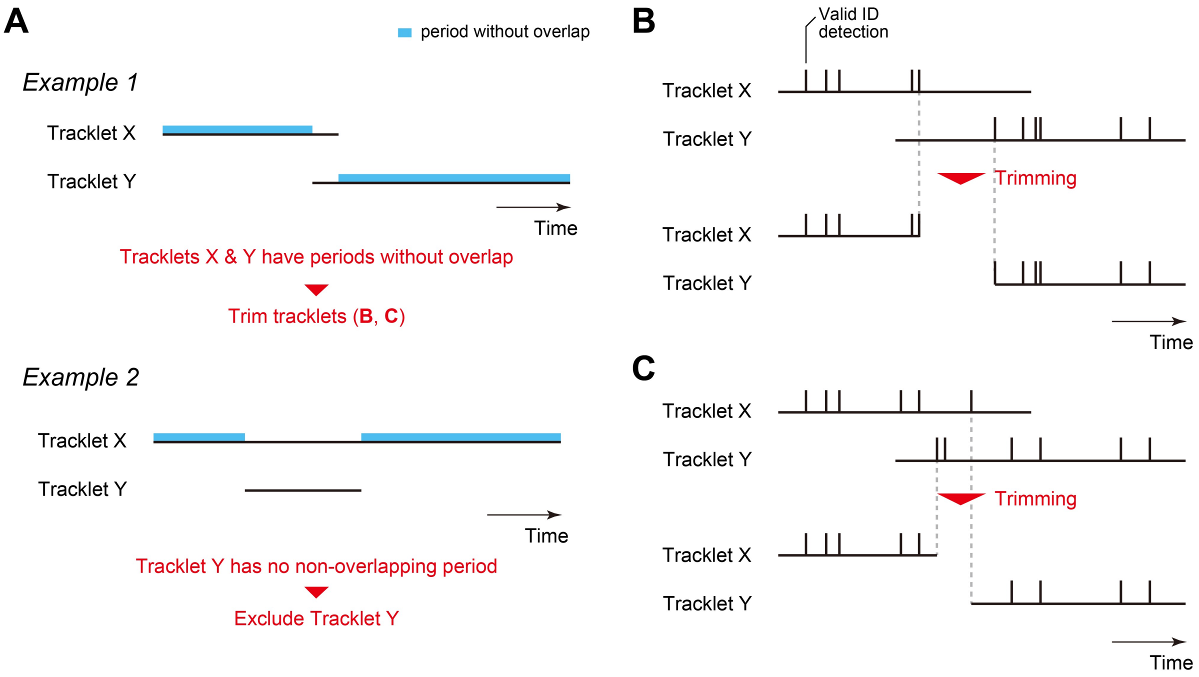

Fig. S8. Resolving temporal overlap of tracklets with the same ID.

(**A**) Overlapping tracklets in which both tracks have a non-overlapping period (light blue area) are trimmed as described in **B** and **C** (Example 1). A tracklet without a non-overlapping period (Tracklet B in Example 2) was excluded. (**B**, **C**) Tracklet trimming. If the last valid ID detection of the preceding tracklet was before the first valid ID detection of the following tracklet, the preceding and following tracklets are trimmed to their own last and first detection time, respectively (**B**). Otherwise, the preceding and following tracklets are trimmed at the other's first and last detection time, respectively (**C**).

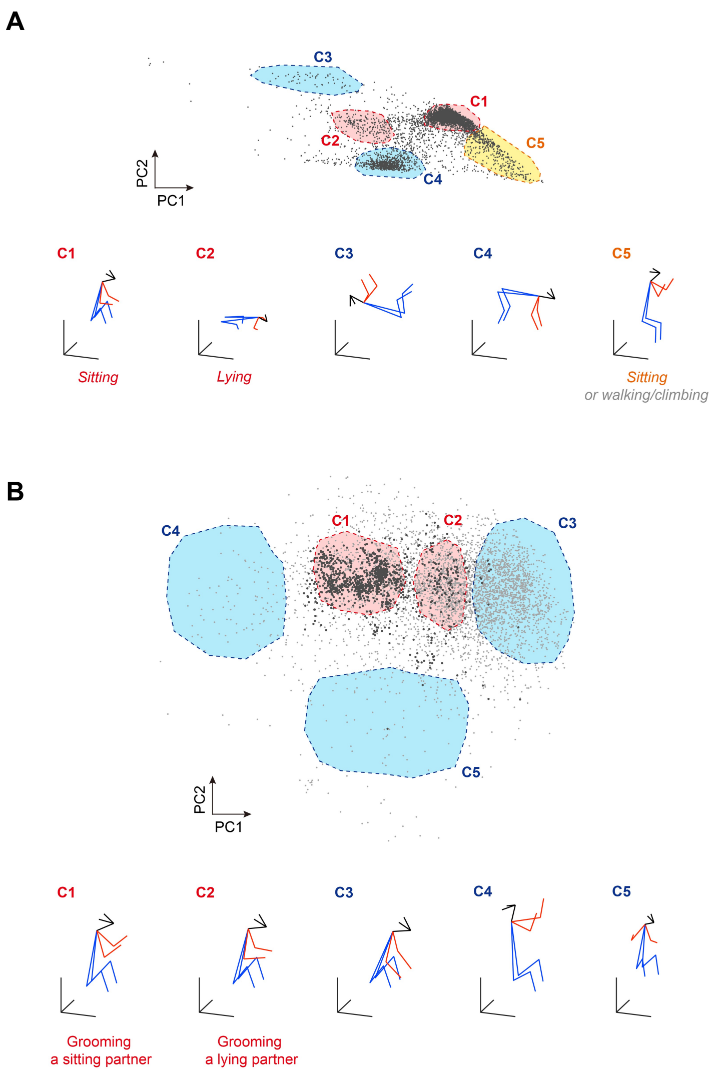

Fig. S9. Classification of postures using principal component analysis (PCA).

(**A**) To classify sitting and lying postures, the vertical positions of keypoints of the lower body (left and right hips, knees, and ankles) are reduced to two dimensions by PCA (top). The boundaries of clusters in the feature space corresponding to sitting (C1, C5) and lying (C2) are drawn for the classification. Since the postures in cluster C5 appear during walking on two legs and climbing a wall, as well as sitting with the legs down, the posture in cluster C5 was classified as sitting only when the hip center is located at a place where a monkey can sit (within 100 mm where the hip center of the postures in cluster C1 are observed). Bottom, visualization of average posture in each cluster. (**B**) To classify postures associated with grooming, 3D positions of keypoints of the upper body (nose, left and right ears, elbows, and wrists; shoulders were not considered since they were fixed in the body-centered coordinate) for monkeys in Proximity are reduced to two dimensions by PCA (top). Some grooming behaviors were manually annotated by visual inspection of videos, and the corresponding postures are plotted with dark dots. From the distribution of the dark plots, the two clusters (C1, C2), corresponding to the postures during grooming for a sitting and lying partner, were identified and used for classification. Bottom, visualization of the average posture of the clusters (C1, C2) and those of the other areas in the feature space (C3–5).

Table S1. Monkey profiles.

Group I

Tag color	Age (years)	Sex	Body weight (kg)	Original troop
Blue	3	Female	4.6	Takahama
Green	4	Female	6.7	Takahama
Red	4	Female	6.1	Arashiyama
White	3	Female	6.1	Takahama

Additional note: Green & White were sisters.

Group II

Tag color	Age (years)	Sex	Body weight (kg)	Original troop
Blue	2	Male	4.0	Wakasa
Green	2	Male	3.5	Wakasa
Red	2	Male	3.6	Wakasa
White	2	Male	4.0	Wakasa

Table S2. Recording sessions.

Group I (late):

	Oct 12	13	15	16	17	18	19	20
08:30–08:35	1	2	-	4	3	2	-	4
09:00–09:05	2	4	-	4	4	4	-	4
09:30–09:35	-	-	-	1	1	-	-	-
10:00–10:05	3	-	-	4	4	4	-	-
10:30–10:35	4	-	-	-	4	-	-	4
11:00–11:05	1	-	-	4	4	-	-	-
11:30–11:35	4	2	-	4	4	4	-	-
12:00–12:05	-	4	4	4	4	-	-	-
12:30–12:35	2	4	-	1	4	1	-	-
13:00–13:05	4	4	3	4	4	-	-	-
13:30–13:35	1	1	3	4	2	-	4	4
14:00–14:05	4	4	-	2	4	-	-	1
14:30–14:35	1	4	-	4	4	-	-	4
15:00–15:05	4	4	-	2	4	-	-	1
15:30–15:35	1	4	-	2	4	-	-	4
16:00–16:05	4	4	-	4	4	-	-	4
16:30–16:35	3	4	-	3	2	4	4	4
17:00–17:05	4	4	-	2	4	-	1	4

1–4, number of monkeys detected for >30 s in the session; -, no recording was conducted, or an experimenter was inside or in front of the cage. The sessions shaded with orange color (with the number of detected monkeys = 4) were used for the behavioral analysis.

No recording was conducted on October 14 due to a technical issue.

Group II:

	Jul 14	15	16	17	18	19	20	21
08:30–08:35	4	4	4	3	4	4	4	4
09:00–09:05	4	4	4	3	2	2	4	4
09:30–09:35	4	-	4	3	4	4	-	4
10:00–10:05	4	4	4	3	4	4	-	3
10:30–10:35	4	4	-	4	4	4	-	4
11:00–11:05	-	-	-	-	4	4	-	4
11:30–11:35	4	4	4	4	4	4	4	4
12:00–12:05	4	4	4	4	3	4	4	4
12:30–12:35	4	4	4	4	4	3	4	4
13:00–13:05	4	4	4	4	4	3	4	4
13:30–13:35	-	4	-	4	4	4	4	4
14:00–14:05	4	4	-	4	4	4	4	4
14:30–14:35	4	4	4	4	4	4	3	4
15:00–15:05	4	3	4	4	3	4	4	4
15:30–15:35	4	4	4	2	4	2	-	4
16:00–16:05	-	4	4	4	4	4	4	4
16:30–16:35	4	4	4	3	2	4	4	4
17:00–17:05	4	4	4	4	4	4	3	4

1–4, number of monkeys detected for >30 s in the session; -, no recording was conducted, or an experimenter was inside or in front of the cage. The sessions shaded with orange color (with the number of detected monkeys = 4) were used for the behavioral analysis.

Group I (early):

	Oct 4	5	6	7	8	9	11
08:30–08:35	-	4	2	3	4	4	-
09:00–09:05	-	3	4	4	2	4	-
09:30–09:35	-	2	-	4	3	1	-
10:00–10:05	-	-	-	2	4	4	4
10:30–10:35	-	4	4	4	4	4	2
11:00–11:05	-	-	-	4	2	4	-
11:30–11:35	-	3	4	-	-	4	-
12:00–12:05	4	-	2	4	4	4	4
12:30–12:35	3	-	4	2	4	3	1
13:00–13:05	1	2	2	1	3	4	4
13:30–13:35	1	2	2	0	2	4	2
14:00–14:05	3	1	4	-	-	1	3
14:30–14:35	-	4	4	-	4	1	4
15:00–15:05	-	3	1	-	1	-	3
15:30–15:35	-	2	4	-	-	-	4
16:00–16:05	1	4	2	-	4	4	2
16:30–16:35	2	1	-	-	-	4	4
17:00–17:05	-	4	3	-	-	4	3

1–4, number of monkeys detected for >30 s in the session; -, no recording was conducted, or an experimenter was inside or in front of the cage. The sessions shaded with orange color (with the number of detected monkeys = 4) were used for the behavioral analysis.
No recording was conducted on October 10 due to a technical issue.

Table S3. Analysis of variance results for Fig 3.

Fig 3C:

	F-value (df_1, df_2)	p-value
Main effect of Date	1.36 (5, 49)	0.26
Main effect of ID	17.7 (2.3, 113.1)	4.5×10^{-8}
Interaction between Date and ID	0.680 (11.5, 113.1)	0.762

df_1, df_2, numerator and denominator degrees of freedom, respectively.

Fig 3D:

	F-value (df_1, df_2)	p-value
Main effect of Date	0.67 (5, 49)	0.65
Main effect of Pair	10.2 (2.3, 113.2)	3.4×10^{-5}
Interaction between Date and Pair	1.23 (11.6, 113.2)	0.275

df_1, df_2, numerator and denominator degrees of freedom, respectively.

Fig 3E:

	F-value (df_1, df_2)	p-value
Main effect of Phase	3.43 (1, 10)	0.094
Main effect of ID	39.9 (3, 30)	1.3×10^{-10}
Interaction between Date and ID	1.14 (3, 30)	0.35

df_1, df_2, numerator and denominator degrees of freedom, respectively.

Fig 3F:

	F-value (df_1, df_2)	p-value
Main effect of Phase	0.0043 (1, 10)	0.95
Main effect of Pair	4.72 (5, 50)	1.3×10^{-3}
Interaction between Phase and Pair	4.95 (5, 50)	9.2×10^{-4}

df_1, df_2, numerator and denominator degrees of freedom, respectively.

Table S4. Filter parameters for detection of social behavioral events.

	I_{max} (s)	D_{min} (s)
Observe	0.04	0.04
Proximity	0.5	0.5
Grooming	1.0	1.0
Mount	1.5	0.17
Chase	0.5	0.25
Glare	1.0	1.0
Grab/Push	2.0	0.08
Pounce	0.5	0.17
Look-to-Face	0.04	0.04

Movie S1. An example of 3D motion capture.

Movie S2. Results of 2D video processing corresponding to Movie S1.

Monkey detections are shown as black rectangles. Pose estimation results are also displayed for each detection. The color of each detection changes to blue, green, red, or white when a corresponding color tag is detected. The number just above the upper left corner of each detection indicates the ID of the single-view tracklet (the IDs across views are unrelated). The number in the upper left corner of each view indicates the ID of the camera. The location of each camera in the cage is shown in the figure at the center of the video.

Movie S3. Examples of Proximity, Groom, Chase, Glare, Grab/Push, Pounce, and Mount events.

Movie S4. Examples of motion sequences classified as Syllable 1 (sitting with a little movement) and 2 (sitting with almost no movement).

Five examples are shown for each syllable. In each example, a white dot appears at the beginning of the syllable and disappears when the syllable ends.

Movie S5. Example use of the custom 3D annotation software.
